# Dual Transcranial Direct Current Stimulation Modulates Hierarchical Functional Network Organization in Post‐Stroke Cognitive Impairment: A Randomized Controlled Trial

**DOI:** 10.1002/cns.71111

**Published:** 2026-09-02

**Authors:** Yao Wang, Wan Liu, Wenjie Yang, Jun Hu, Xinxin Cheng, Hao Yu, Yin Shi, Li Liu, Jiang Rao, Guangxu Xu

**Affiliations:** ^1^ Department of Rehabilitation Medicine The Affiliated Brain Hospital of Nanjing Medical University Nanjing Jiangsu Province China; ^2^ Department of Radiology The Affiliated Brain Hospital of Nanjing Medical University Nanjing Jiangsu Province China; ^3^ Institution of Brain Functional Imaging Nanjing Medical University Nanjing Jiangsu Province China; ^4^ Department of Biostatistics, School of Public Health Nanjing Medical University Nanjing Jiangsu Province China; ^5^ Department of Radiology The First Affiliated Hospital of Nanjing Medical University Nanjing Jiangsu Province China; ^6^ Rehabilitation Medicine Center The First Affiliated Hospital of Nanjing Medical University Nanjing Jiangsu Province China

**Keywords:** cognitive impairment, functional magnetic resonance imaging, stroke, transcranial direct current stimulation

## Abstract

**Objective:**

To evaluate the clinical efficacy of dual transcranial direct current stimulation (tDCS) in patients with post‐stroke cognitive impairment (PSCI) and to explore the effects on the hierarchical organization of functional brain networks, ranging from regional synchronization to inter‐regional connectivity and global network topology.

**Methods:**

In this randomized, double‐blind, sham‐controlled trial, 74 PSCI patients received conventional therapy alongside either active dual‐tDCS (*n* = 38) or sham stimulation (*n* = 36). Active tDCS targeted the dorsolateral prefrontal cortex (DLPFC) via anodal‐left/cathodal‐right nodes (2.0 mA, 20 min/day, 20 sessions). The primary outcome was the Montreal Cognitive Assessment (MoCA). Secondary outcomes included the Mini‐Mental Status Examination (MMSE), Stroop Test (ST), Trail Making Test (TMT), Wechsler Memory Scale (WMS), and Barthel Index (BI). A subgroup of 36 participants (18 per group) underwent resting‐state functional magnetic resonance imaging (rs‐fMRI) to analyze regional homogeneity (ReHo), functional connectivity (FC), and network topology. Partial correlations assessed the association between neuroimaging alterations and clinical improvements.

**Results:**

The tDCS group showed significantly greater improvements in MoCA scores (tDCS: 5.74 ± 2.76 vs. sham: 2.69 ± 2.69; *t* = 4.799, *p* < 0.001) as well as in attention and memory domains compared to the sham group. The rs‐fMRI changes included increased ReHo in the right middle temporal gyrus (MTG) and the left inferior frontal gyrus (IFG), and reduced FC between the right MTG‐left superior frontal gyrus and left IFG‐cerebellum (*p* < 0.05, FWE‐corrected). Additionally, small‐worldness and global efficiency increased (*p* < 0.05) with these alterations correlating with clinical recovery. Adverse events were rare and self‐limiting.

**Conclusion:**

Dual‐tDCS over bilateral DLPFC safely improves cognitive recovery in PSCI. These clinical gains are associated with rs‐fMRI alterations, specifically in regional synchronization, inter‐regional connectivity, and global topology, which suggest a potential biomarker for monitoring tDCS efficacy, offering a rationale for precision neuromodulation in stroke rehabilitation.

## Graphical abstract

Bilateral DLPFC dual‐tDCS significantly promotes cognitive recovery by modulating the rs‐fMRI hierarchical organization of functional brain networks, ranging from regional synchrony, distant regions connectivity and global network topological integration. These network changes correlate with clinical gains, providing a promising neuroimaging biomarker and a solid rationale for precision neuromodulation.

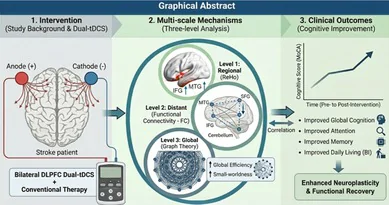

## 1 Introduction

Stroke remains a leading cause of global disability, with post‐stroke cognitive impairment (PSCI) representing a debilitating sequela that significantly elevates the risk of post‐stroke dementia (PSD), imposing a substantial socio‐economic burden [1, 2]. Despite its prevalence, effective interventions remain limited. While pharmacological treatments (e.g., cholinesterase inhibitors) are commonly used, their efficacy in PSCI is often modest and limited by potential side effects [3]. Similarly, although non‐pharmacological cognitive training is a cornerstone of rehabilitation, its impact is frequently constrained by therapist‐dependent variability and suboptimal patient engagement [2]. Thus, there is an urgent need for adjunctive, evidence‐based neurorehabilitation strategies.

Transcranial direct current stimulation (tDCS) has emerged as a promising non‐invasive brain stimulation (NIBS) modality. By delivering a weak constant current to modulate cortical excitability, tDCS has shown potential in enhancing cognitive functions across various neurological populations [4]. In PSCI specifically, conventional unilateral anodal stimulation over the left dorsolateral prefrontal cortex (DLPFC) has been the primary focus; however, clinical outcomes remain inconsistent across studies [5, 6, 7]. Emerging evidence suggests that dual‐tDCS (simultaneous anodal/cathodal stimulation) may yield superior efficacy by modulating bilateral hemispheric balance [8, 9, 10], yet high‐quality randomized controlled trials (RCTs) clarifying its clinical utility in PSCI are still lacking.

To understand how tDCS facilitates recovery, resting‐state functional magnetic resonance imaging (rs‐fMRI) has been employed to investigate alterations in functional network organization. Previous studies have identified regional homogeneity (ReHo) or functional connectivity (FC) alterations in PSCI versus healthy control or following NIBS neuromodulation; the current literature focusing on the synergy of tDCS and fMRI in PSCI remains sparse and fragmented [11, 12, 13, 14, 15]. Some research ignored regional synchrony with poorly defined stimulation protocols [13], others focused predominantly on a single analytical level of connectivity, thereby failing to capture the hierarchical network alterations that integrate regional synchronization with global network topology [14]. Crucially, few studies have integrated the hierarchical organization of functional brain networks across local, inter‐regional, and global topological levels, meaning that network‐level alterations during tDCS‐mediated recovery warrant further characterization.

Therefore, this randomized, double‐blind, sham‐controlled study aimed to evaluate the therapeutic efficacy of dual‐tDCS in PSCI and to characterize its effects across distinct rs‐fMRI metrics. We hypothesized that dual‐tDCS would induce superior cognitive gains that would be associated with specific functional network alterations, serving as a potential biomarker for monitoring treatment efficacy. The novelty of this study lies in its integrated approach, bridging regional synchrony, distant connectivity, and global network topology to establish a robust rationale for monitoring and understanding post‐stroke functional alterations induced by neuromodulation.

## 2 Materials and Methods

### 2.1 Study Design and Randomization

This prospective, single‐center, randomized, double‐blind, sham‐controlled trial was registered with the Chinese Clinical Trial Registry (ChiCTR‐2200055500, Registration Date: January 11th, 2022) and was reported in accordance with the Consolidated Standards of Reporting Trials (CONSORT) 2010 statement (Data S1). Participants were randomized 1:1 into the active tDCS or sham group using a computer‐generated sequence managed by an independent statistician. Allocation concealment was maintained using sealed, opaque envelopes. Patients, neuropsychological assessors, and neuroimaging data analysts were unaware of the group assignments. Blinding efficacy was assessed via a post‐intervention questionnaire.

The formula for sample size was *n*_1_ = *n*_2_ = [2(*u*_α_ + *u*_β_)^2^ *σ* ^2^]/*δ* ^2^. Based on a literature‐informed effect size (*δ* = 2.90, *σ* = 3.18) with *α* = 0.05 and *β* = 0.10, a minimum of 31 subjects per group was required. Accounting for a 10% attrition rate, we recruited 70 patients (35 per group).

### 2.2 Participants

Patients with post‐stroke cognitive impairment (PSCI) were recruited from the Department of Rehabilitation Medicine, Nanjing Medical University affiliated Brain Hospital (April 2022–December 2024).

Inclusion criteria: (1) Age 50–80 years; (2) First‐ever stroke (CT/MRI confirmed); (3) Cognitive impairment persisting > 3 months post‐stroke; (4) Right‐handed; (5) Mini‐Mental Status Examination (MMSE) thresholds (Illiteracy < 17, Primary < 20, Junior high+ < 24) [16].

**FIGURE 1:**
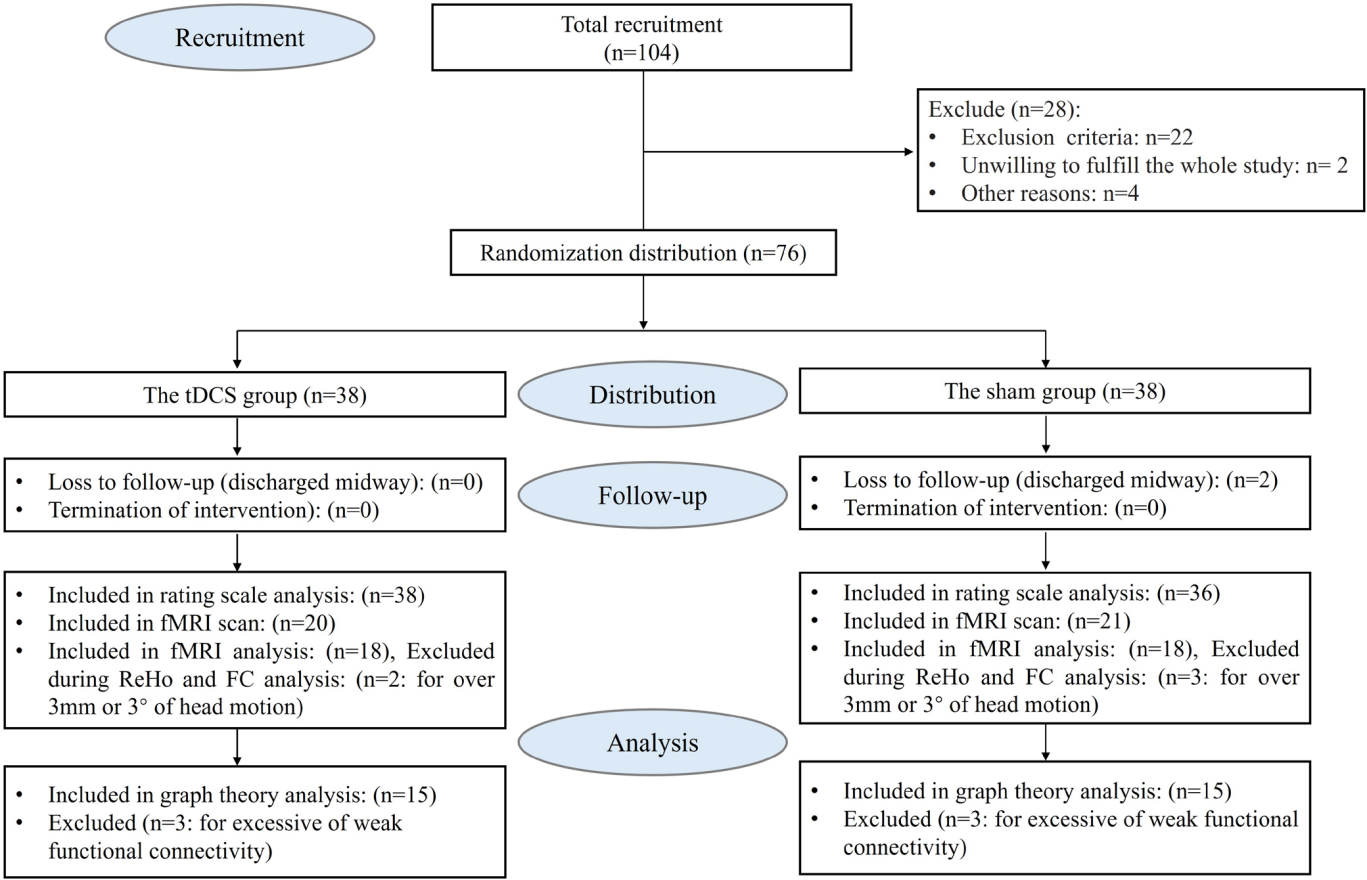
The enrollment process for this study. FC: Functional connectivity; fMRI: Functional Magnetic Resonance Imaging; ReHo: Regional homogeneity; tDCS: Transcranial Direct Current Stimulation.

Exclusion criteria: (1) Pre‐existing cognitive impairment, dementia, other neurodegenerative diseases, or dementia family history; (2) Hachinski Ischemic Score < 4; (3) Fazekas scores ≥ 2; (4) Major systemic illness or metallic implants; (5) Severe sensory/language deficits; (6) History of epilepsy or prior tDCS; (7) Pregnancy/lactation. Enrollment details are illustrated in Figure 1.

### 2.3 Intervention Protocol

All participants received standard medication (e.g., anti‐platelet agents, statins, and neuroprotective drugs) without significant changes in types or dosages during the whole intervention period and conventional rehabilitation by experienced therapists (30 min/session, 5 days/week for 4 weeks). The detailed medication and training components were clarified in the Data S1.

tDCS Parameters: Delivered via an 8060 Smart Electrical Stimulator (Wuhan Yimai Medical Technology Co. Ltd.) employing 3.5 × 3.5 cm^2^ saline‐soaked sponge electrodes. A 2.0 mA current (density: 0.057 mA/cm^2^) was applied for 20 min/session per day, 5 days/week for 4 weeks (20 sessions total). The anode was at F3 and the cathode at F4 according to the EEG hat [10].

Sham Protocol [17]: Identically electrodes placement, but the stimulator was ramped down after the first 30 s to simulate initial sensations. The device's appearance and interface were identical to the active mode.

### 2.4 Assessment

Primary Outcome: Global cognitive function: Montreal Cognitive Assessment (MoCA) [18]; Secondary Outcomes: (1) MMSE; (2) Attention function: Stroop Test (ST) Parts A and B completion time [19]; (3) Executive function: ST Part C and Trail Making Test (TMT) completion time [20]; (4) Memory function: Wechsler Memory Scale (WMS) [21]; (5) Activities of daily living: Barthel Index (BI). Adverse events were recorded at each session. Assessments occurred < 3 days before and after the 4‐week intervention.

### 2.5 fMRI Acquisition and Preprocessing

MRI data were acquired using a 3.0T scanner (Magnetom Verio, Siemens Healthcare, Erlangen, Germany) with a 12‐channel head coil.

For functional analysis and localization, we acquired high‐resolution T1‐weighted structural images (3D MPRAGE; TR/TE = 1900/2.48 ms, TI = 900 ms, isotropic voxel size = 1.0 mm^3^, 176 slices) and BOLD functional images (EPI; TR/TE = 2000/30 ms, voxel size = 3.4 × 3.4 × 4.0 mm^3^, 240 volumes).

For routine clinical assessment, T2‐weighted FLAIR images were obtained (TR/TE = 8000/81 ms, TI = 2373.3 ms). To ensure the reliability of the Fazekas scale for white matter hyperintensity assessment, images were evaluated by two independent neuroradiologists. Other routine sequences, including T2‐weighted images (T2WI) and diffusion‐weighted images (DWI), were also performed to exclude organic brain lesions. Detailed sequences are in Data S1 consistent with our earlier research [15].

Data Processing Assistant for Resting‐State fMRI (DPARSF; http://www.restfmri.net/forum/DPARSF) was employed to preprocess data utilizing Statistical Parametric Mapping (SPM8) (http://www.fil.ion.ucl.ac.uk/spm) in the MATLAB environment (Mathworks, Natick, MA, USA). Preprocessing steps included: (1) Removal of the first 10 volumes; (2) Slice‐timing and realignment (exclusion for cumulative translation or rotation > 3.0 mm or 3°) [22]; (3) Normalized to standard Montreal Neurological Institute (MNI) space using DARTEL, resampled to a 3 × 3 × 3 mm^3^ voxel size; (4) Regression of 24 motion parameters, global signal, white matter, and cerebrospinal fluid signals; (5) Band‐pass filtering (0.01–0.08 Hz).

### 2.6 Imaging Data Analysis

ReHo: Kendall's coefficient of concordance (KCC) on unsmoothed data across 26 neighboring voxels. Individual ReHo maps were normalized to the global mean and then smoothed (6 mm FWHM) for group analysis [23].

FC: Significant ReHo clusters were defined as regions of interest (ROIs). Seed‐based FC used Fisher's *r*‐to‐*z* transformed correlation maps [24].

Graph Theory: Nodes were defined by the AAL‐116 atlas (Figure S1). Edges were constructed via partial correlations of regional time series. Binary adjacency matrices were generated over a sparsity range (0.0414–0.2514, interval 0.01) where small‐worldness *σ* > 1.1 [25]. Global network metrics such as small‐world parameters [26] (clustering coefficient *C*_p_, characteristic path length *L*_p_, normalized clustering coefficient *γ*, normalized characteristic path length *λ*, and small‐worldness *σ*) and network efficiency [25] (local efficiency *E*_loc_ and global efficiency *E*_glob_) were calculated using the GRETNA toolbox (http://www.nitrc.org/projects/gretna/) with Area Under the Curve (AUC) used for threshold‐independent comparison [27, 28].

### 2.7 Correlation

Partial correlation analyses assessed the relationship between the longitudinal imaging measures (ReHo, FC, and network topological features) and improvements in neuropsychological test scores, entering age, sex, years of education, lesion volume, disease duration, stroke type, and baseline National Institute of Health stroke scale (NIHSS)/Fazekas scores as covariates.

### 2.8 Statistical Analysis

Statistical procedures were performed in SPSS 25.0. Continuous baseline data were compared via one‐way analysis of variance (ANOVA) or Mann–Whitney *U* tests, and categorical variables via *χ* ^2^ tests. Group × time interactions for clinical outcomes and imaging/network metrics were analyzed using repeated‐measures ANOVA, followed by post hoc *t*‐tests [29, 30].

For fMRI, significance was set at voxel‐level *p* < 0.001 and cluster‐level *p* < 0.05, family‐wise error (FWE) corrected [31, 32]. Partial correlation analyses evaluated the relationship between imaging alterations and clinical recovery, with false discovery rate (FDR) correction applied for multiple comparisons. *p* < 0.05 was considered statistically significant.

## 3 Results

### 3.1 Clinical Characteristics

Seventy‐four participants completed the intervention and neuropsychological evaluations (tDCS: *n* = 38; sham: *n* = 36), with no significant inter‐group differences in baseline demographics or clinical scales (*p* > 0.05; Table 1). While all 74 participants underwent baseline fMRI, longitudinal post‐intervention imaging was obtained from 41 participants (tDCS: *n* = 20; sham: *n* = 21) due to funding constraints. Five cases were excluded for exceeding the head motion threshold (> 3 mm or 3°), leaving a subset of 36 participants (tDCS: *n* = 18; sham: *n* = 18; Figure 1). This subset showed balanced baseline characteristics, including Fazekas scores (*p* > 0.05; Table S1). Lesion distribution maps of the participants are illustrated in Figure S2.

**TABLE 1:** Characteristics at baseline for the tDCS and the sham groups.

Characteristic	tDCS group	Sham group	*p*
	(*n* = 38)	(*n* = 36)	
Demographics			
Gender (male/female), *n*	26/12	28/8	0.365
Age (years), Mean ± SD	61.42 ± 13.45	61.08 ± 11.66	0.909
Education, *n*			0.933
≤ 9 years	18	17	
> 9 years	20	19	
Stroke history			
Duration (days), Mean ± SD	195.00 ± 243.14	226.78 ± 229.16	0.617
Stroke type (ischemia/hemorrhage), *n*	23/15	22/14	0.959
Side of lesion (left/right), *n*	19/19	19/17	0.811
Site of lesion, *n*			0.778
Cortico‐subcortical strokes	19	20	
Deep hemispheric WM and GM strokes	10	7	
Brainstem/cerebellar strokes	9	9	
NIHSS level, Median (IQR)^a^	3.00 (3.00–3.25)	3.00 (3.00–3.00)	0.674
Baseline cognitive status			
MoCA Mean ± SD	13.24 ± 4.47	14.47 ± 4.64	0.247
MMSE, Mean ± SD	18.63 ± 5.70	19.83 ± 4.76	0.329
ST‐A (seconds), Mean ± SD	68.34 ± 13.72	67.92 ± 14.65	0.898
ST‐B (seconds), Mean ± SD	86.39 ± 15.72	86.61 ± 16.00	0.953
ST‐C (seconds), Mean ± SD	107.50 ± 15.45	107.17 ± 18.75	0.934
TMT (seconds), Mean ± SD	260.47 ± 95.86	259.69 ± 92.37	0.972
WMS, Mean ± SD	65.61 ± 17.95	66.50 ± 12.76	0.806
BI, Mean ± SD	56.71 ± 21.03	57.78 ± 18.22	0.817

Abbreviations: BI: Barthel Index; GM: Gray Matter; IQR: Interquartile Range; MMSE: Mini‐Mental Status Examination; MoCA: Montreal Cognitive Assessment; NIHSS: National Institute of Health stroke scale; SD: Standard Deviation; ST: Stroop Test; tDCS: Transcranial Direct Current Stimulation; TMT: Trail Making Test; WMS: Wechsler Memory Scale; WM: White Matter.

^a^ NIHSS scores were categorized into 5 levels: Level 1 (0–1), Level 2 (2–4), Level 3 (5–15), Level 4 (16–20), and Level 5 (≥ 21).

### 3.2 Neuropsychological Outcomes

#### 3.2.1 Primary Outcomes

**FIGURE 2:**
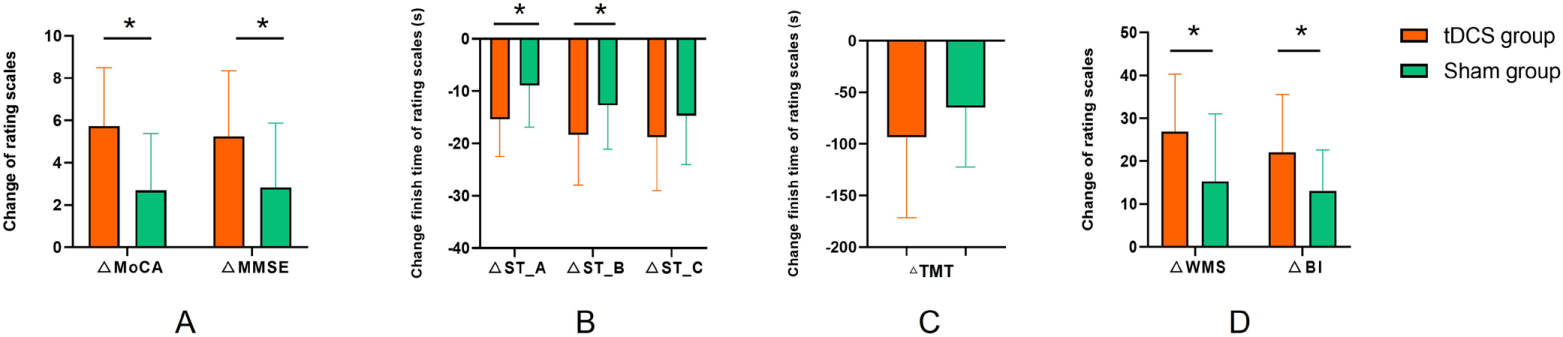
Group × time interaction effects of rating scales following dual‐tDCS. (A) MoCA and MMSE scores, (B) Total completion time for ST‐A/B/C; (C) Total completion time for TMT; (D) WMS and BI scores. BI: Barthel Index; MMSE: Mini‐Mental Status Examination; MoCA: Montreal Cognitive Assessment; ST: Stroop Test; tDCS: Transcranial Direct Current Stimulation; TMT: Trail Making Test; WMS: Wechsler Memory Scale; *p < 0.05.

MoCA scores demonstrated a significant group × time interaction (*F* = 4.677, *p* = 0.034, $\eta_{p}^{2}$ = 0.061). The tDCS group exhibited greater increases compared to sham (tDCS: 5.74 ± 2.76 vs. sham: 2.69 ± 2.69; *t* = 4.799, *p* < 0.001; Figure 2A).

#### 3.2.2 Secondary Outcomes

Group × time interaction effects were significant for MMSE (*F* = 11.244, *p* = 0.001, $\eta_{p}^{2}$ = 0.135), ST‐A (*F* = 12.278, *p* = 0.001, $\eta_{p}^{2}$ = 0.146), ST‐B (*F* = 5.749, *p* = 0.019, $\eta_{p}^{2}$= 0.074), WMS (*F* = 11.022, *p* = 0.001, $\eta_{p}^{2}$ = 0.133), and BI (*F* = 11.016, *p* = 0.001, $\eta_{p}^{2}$ = 0.133). The tDCS group showed larger increases compared to sham in MMSE (tDCS: 5.24 ± 3.11 vs. sham: 2.83 ± 3.05; *t* = 3.353, *p* = 0.001), WMS (tDCS: 26.84 ± 13.50 vs. sham: 15.28 ± 15.74; *t* = 3.399, *p* = 0.001), and BI (tDCS: 22.11 ± 13.44 vs. sham: 13.06 ± 9.58; *t* = 3.319, *p* = 0.001; Figure 2A,D), as well as greater reductions in completion times for ST‐A (tDCS: −15.39 ± 7.13 s vs. sham: −8.94 ± 7.90 s; *t* = −3.692, *p* < 0.001) and ST‐B (tDCS: −18.37 ± 9.61 s vs. sham: −12.67 ± 8.41 s; *t* = −2.710, *p* = 0.008; Figure 2B). No significant interactions were observed for ST‐C or TMT (*p* > 0.05).

### 3.3 fMRI Findings

#### 3.3.1 ReHo And Seed‐Based FC

**FIGURE 3:**
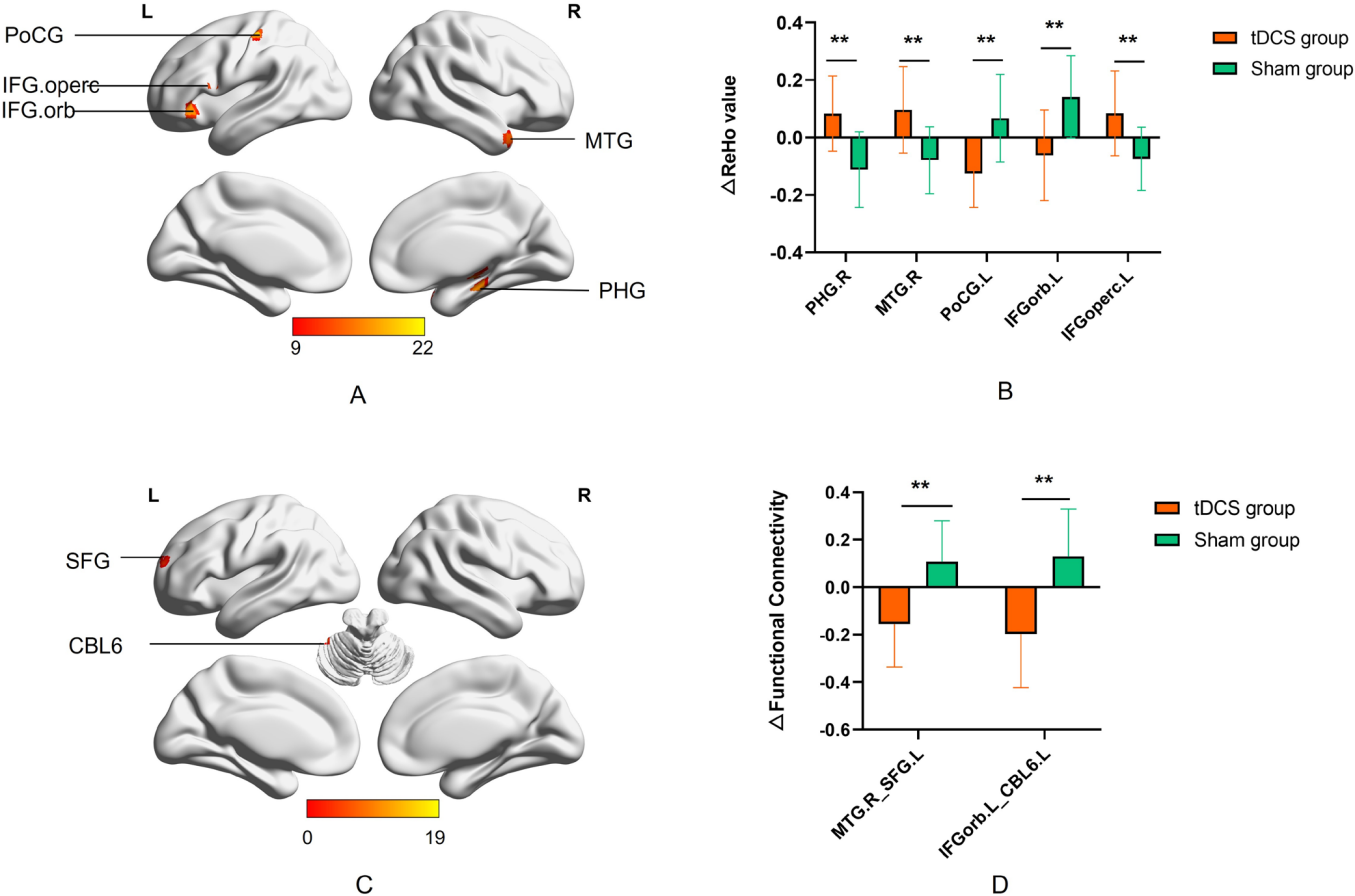
Significant group × time interaction effects on ReHo and FC following dual‐tDCS. (A) Significant interaction effects on ReHo located in the right PHG (MNI: 21, −21, −18), MTG (MNI: 48, 15, −30), left IFGoperc (MNI: −60, 12, 18), left PoCG (MNI: 54, −21, 60), and left IFGorb (MNI: −42, 30, −6); (B) Mean ReHo values for each group pre‐ and post‐treatment; (C) Significant interaction effects on FC in connections of right MTG‐left SFG (MNI: −18, 57, 18) and left IFGorb‐CBL6 (MNI: −42, −39, 30); (D) Mean FC values for each group pre‐ and post‐treatment. Repeated measures ANOVA method, brain regions with significant group × time interaction effect, multiple comparison correction used voxel level *p* < 0.001, cluster level *p* < 0.05, FWE correction, cluster size threshold = 10. Anatomical labels were assigned using the Anatomical Automatic Labeling (AAL) atlas. CBL6: Cerebellar lobule VI; FC: Functional connectivity; IFG.operc: Inferior frontal gyrus opercular part; IFG.orb: Inferior frontal gyrus orbital part; L: Left; MTG: Middle temporal gyrus; PHG: Parahippocampal gyrus; PoCG: Postcentral gyrus; R: Right; ReHo: Regional homogeneity; SFG: Superior frontal gyrus; tDCS: Transcranial Direct Current Stimulation; **p < 0.001.

Significant interactions in ReHo were identified within the left inferior frontal gyrus (IFG), postcentral gyrus (PoCG), right parahippocampal gyrus (PHG), and middle temporal gyrus (MTG) (*p* < 0.05, FWE‐corrected; Figure 3A, Table 2). Specifically, post hoc analyses within these clusters showed that ReHo increased in the right PHG/MTG and left IFGoper, whereas it decreased in the left PoCG/IFGorb in the tDCS group (*p* < 0.05; Figure 3B).

**TABLE 2:** The brain regions of significant group × time interaction effects of ReHo and FC following dual‐tDCS.

Brain region (AAL)	Peak MNI coordinate			*F*	Cluster size
	*X*	*Y*	*Z*		
ReHo					
Right parahippocampal gyrus	21	−21	−18	16.44	15
Right middle temporal gyrus	48	15	−30	14.07	14
Left inferior frontal gyrus opercular part	−60	12	18	13.83	10
Left postcentral gyrus	54	−21	60	21.08	14
Left inferior frontal gyrus orbital part	−42	30	−6	16.28	12
FC					
Left superior frontal gyrus (Right middle temporal gyrus as ROI)	−18	57	18	18.63	11
Left cerebellar lobule VI (Left inferior frontal gyrus as ROI)	−42	−39	−30	18.82	13

*Note:* Repeated measures ANOVA method, brain regions with significant group × time interaction effect, multiple comparison correction used voxel level *p* < 0.001, cluster level *p* < 0.05, FWE correction, cluster size threshold = 10. AAL: Automated Anatomical Labeling; FC: Functional connectivity; ReHo: Regional homogeneity; tDCS: Transcranial direct current stimulation; MNI: Montreal Neurological Institute.

Seed‐based FC analysis revealed significant interactions for right MTG‐left SFG and left IFGorb‐cerebellar lobule VI (CBL6) connections (*p* < 0.05, FWE‐corrected; Figure 3C, Table 2). Post hoc analyses comparisons indicated that FC strength in these two connections decreased in the tDCS group compared to the sham group (*p* < 0.05; Figure 3D).

#### 3.3.2 Graph Theoretical Analysis

**FIGURE 4:**
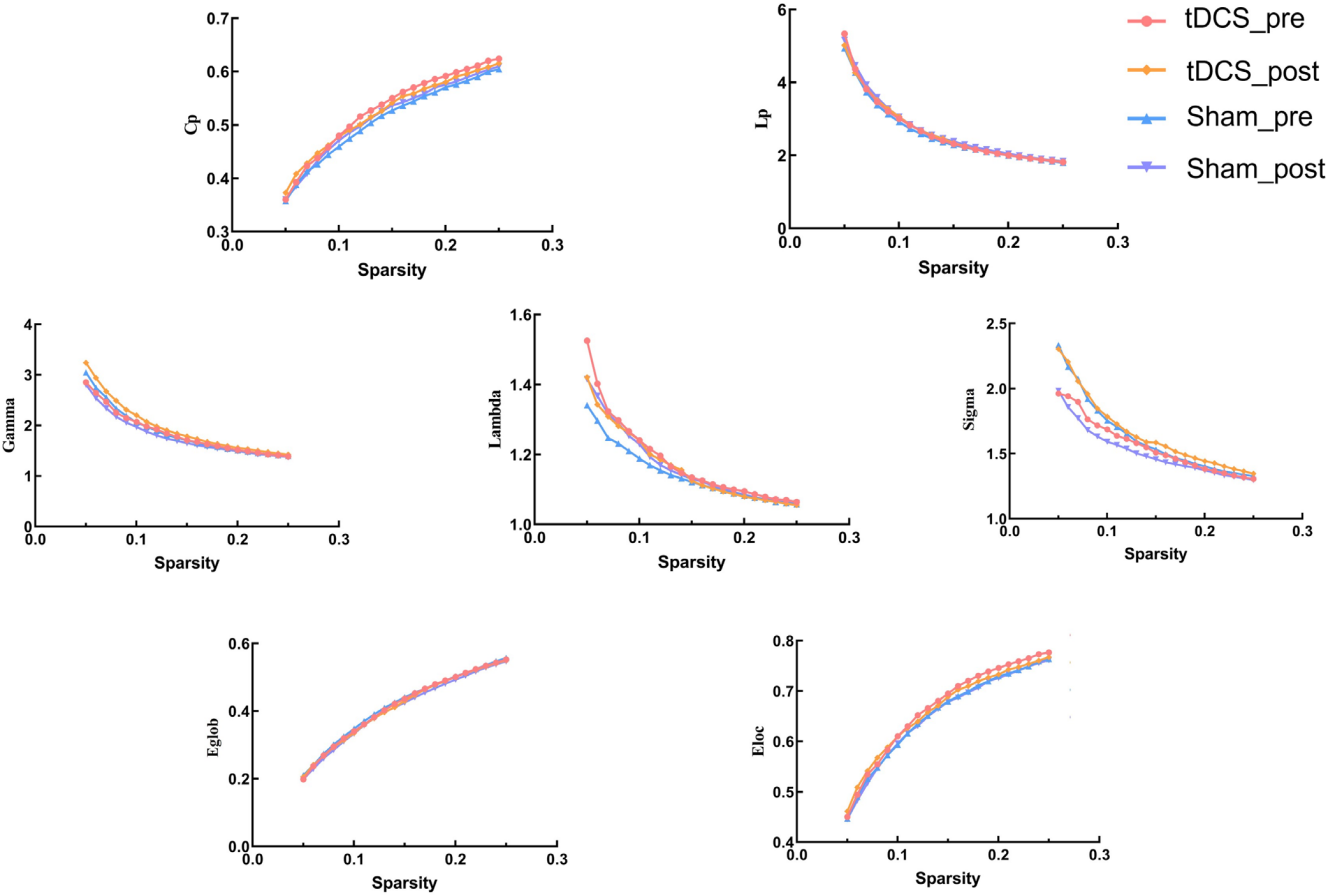
Distribution of global properties metrics for each group under different sparsity levels. *C*_p_: Clustering coefficient; *E*_glob_: Global efficiency; *E*_loc_: Local efficiency; Gamma (γ): Normalized clustering coefficient; Lambda (*λ*): Normalized characteristic path length; *L*_p_: Characteristic path length; Sigma (*σ*): Small‐worldness; tDCS: Transcranial Direct Current Stimulation.

For this analysis, 30 participants (tDCS: *n* = 15; sham: *n* = 15) were included. Six participants were excluded (tDCS: *n* = 3; sham: *n* = 3) because their functional connectivity matrices failed to meet the small‐worldness criterion (*σ* > 1.1) across the predefined sparsity range, indicating possible topological instability. Figure 4 presents the distributions of network metrics at varying levels of sparsity.

**FIGURE 5:**
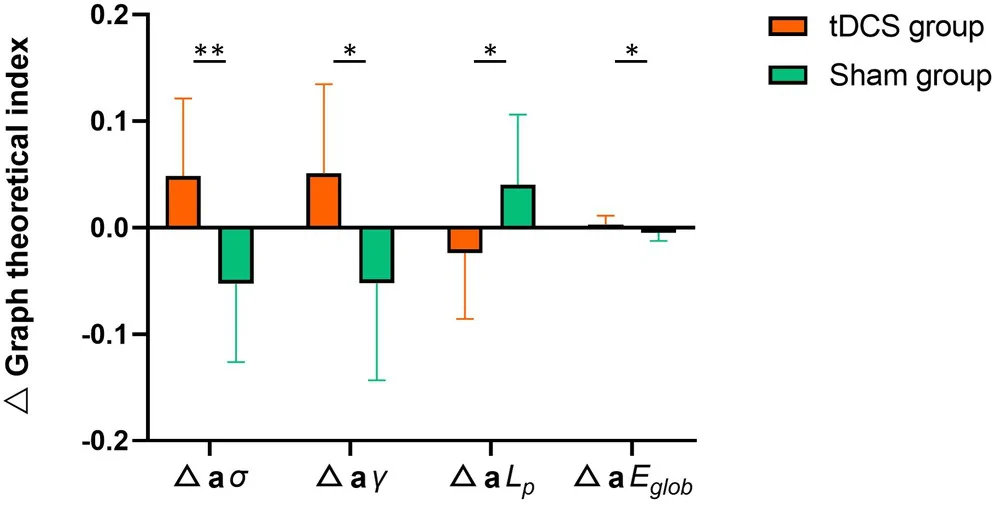
Group × time interaction effects of graph theoretical analysis following dual‐tDCS. *E*_glob_: Global efficiency; Gamma (*γ*): Normalized clustering coefficient; *L*_p_: Characteristic path length; Sigma (*σ*): Small‐worldness; tDCS: Transcranial Direct Current Stimulation.*p < 0.05, **p < 0.001.

Significant interactions were found for small‐worldness (*σ*: *F* = 14.367, *p* = 0.001, $\eta_{p}^{2}$ = 0.339), normalized clustering coefficient (*γ*: *F* = 10.434, *p* = 0.003, $\eta_{p}^{2}$ = 0.271), characteristic path length (*L*_p_: *F* = 7.552, *p* = 0.010, $\eta_{p}^{2}$ = 0.212), and global efficiency (*E*_glob_: *F* = 6.356, *p* = 0.018, $\eta_{p}^{2}$ = 0.185; Table 3). Compared to the sham group, the tDCS group exhibited significantly larger increases in *σ* (tDCS: 0.04851 ± 0.07252 vs. sham: −0.05266 ± 0.07366; *t* = 3.790, *p* = 0.001), *γ* (tDCS: 0.05086 ± 0.08359 vs. sham: −0.05226 ± 0.09111; *t* = 3.230, *p* = 0.003), and *E*_glob_ (tDCS: 0.00260 ± 0.00851 vs. sham: −0.00484 ± 0.00763; *t* = 2.521, *p* = 0.018), alongside a greater reduction in *L*_p_ (tDCS: −0.02385 ± 0.06193 vs. sham: 0.04021 ± 0.06571; *t* = −2.748, *p* = 0.01; Figure 5).

**TABLE 3:** Group × time interaction effects of graph theoretical analysis following dual‐tDCS.

Global measures	*F*	*p*
Clustering coefficient, *C*_p_	0.355	0.556
Characteristic path length, *L*_p_	7.552	**0.010** *
Normalized clustering coefficient, *γ*	10.434	**0.003** *
Normalized characteristic path length, *λ*	3.902	0.058
Small‐worldness, *σ*	14.367	**0.001** *
Global efficiency, *E*_glob_	6.356	**0.018** *
Local efficiency, *E*_loc_	1.626	0.213

*Note:* Bold text indicates results with statistically significant differences.

* *p* < 0.05.

### 3.4 Correlation Analysis

**FIGURE 6:**
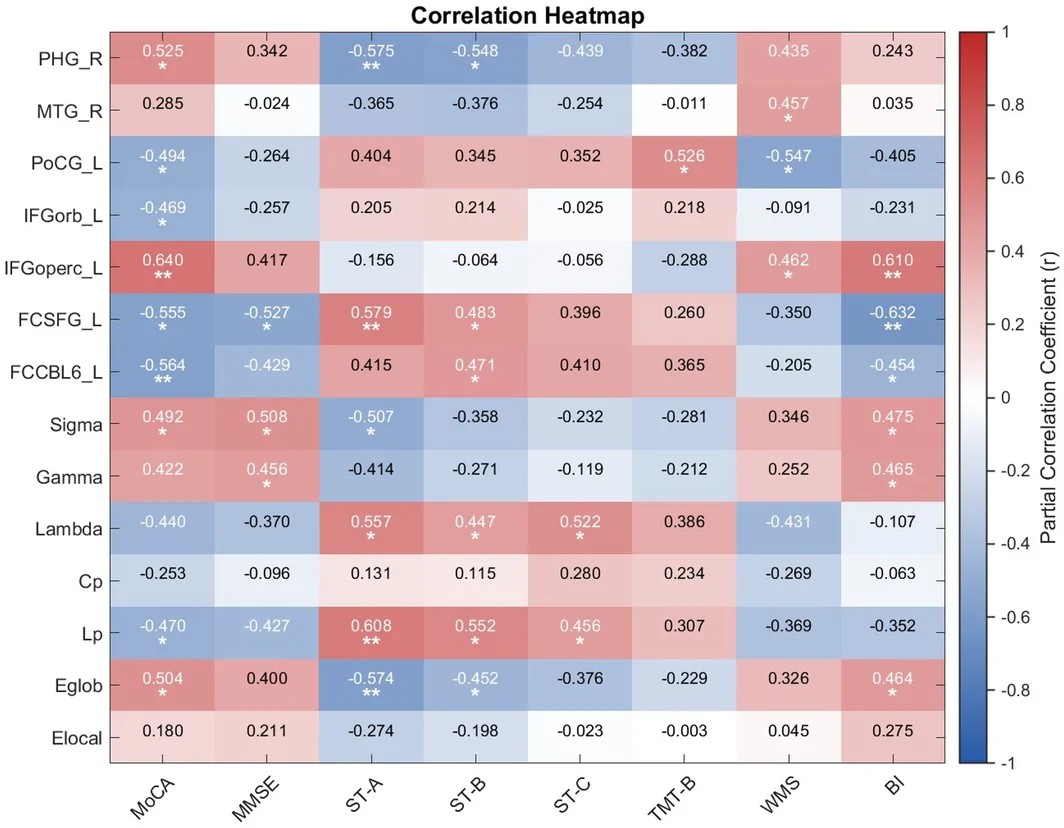
Heatmap about partial correlation regarding change of clinical/cognitive scores and fMRI measurements. All correlation analyses were adjusted for age, sex, education, stroke type, disease duration, lesion volume, baseline NIHSS/Fazekas scores as covariates. The color bar on the right indicates the strength and direction of the correlation, with *r* values explicitly annotated within each cell. BI: Barthel Index; *C*_p_: clustering coefficient; *E*_glob_: global efficiency; *E*_loc_: local efficiency; FCCBL: functional connectivity of left cerebellar lobule VI and left inferior frontal gyrus; FCSFG: functional connectivity of left superior frontal gyrus and right middle temporal gyrus; Gamma (*γ*): normalized clustering coefficient; IFG.operc: inferior frontal gyrus opercular part; IFG.orb: inferior frontal gyrus orbital part; Lambda (*λ*): normalized characteristic path length; *L*_p_: characteristic path length; MMSE: Mini‐Mental Status Examination; MoCA: Montreal Cognitive Assessment; MTG: middle temporal gyrus; PHG: parahippocampal gyrus; PoCG: postcentral gyrus; Sigma (*σ*): small‐worldness; ST: Stroop Test; TMT: Trail Making Test; WMS: Wechsler Memory Scale. **p < 0.001, *p **<** 0.05 after False Discovery Rate (FDR) correction.

Partial correlation analyses demonstrated that changes (△) in clinical scores were significantly associated with neuroimaging interactions (*p* < 0.05, FDR‐corrected). Specifically, △MoCA positively correlated with △ReHo in the right PHG (*r* = 0.525, *p* = 0.017) and left IFGoperc (*r* = 0.640, *p* = 0.002), as well as △*σ* (*r* = 0.492, *p* = 0.028) and △*E*_glob_ (*r* = 0.504, *p* = 0.024). Conversely, △MoCA was negatively associated with △ReHo in the left PoCG (*r* = −0.494, *p* = 0.027) and left IFGorb (*r* = −0.469, *p* = 0.037), △FC of the right MTG‐left SFG (*r* = −0.555, *p* = 0.011) and the left IFGorb‐left CBL6 (*r* = −0.564, *p* = 0.010), and △*L*_p_ (*r* = −0.470, *p* = 0.037). Additional correlation patterns are summarized in the heatmap (Figure 6).

### 3.5 Safety

tDCS was well‐tolerated, with mild adverse effects (tingling, erythema, or itching) reported by three participants when stimulating during the first sessions, which resolved immediately after the session without intervention. No serious adverse events or withdrawals occurred.

## 4 Discussion

This randomized, double‐blind, sham‐controlled trial demonstrates that bilateral DLPFC dual‐tDCS significantly enhances global cognition, attention, memory, and activities of daily living (ADL) in patients with PSCI. By integrating different analytical resting‐state fMRI metrics, we characterize a hierarchical pattern of functional network alterations: dual‐tDCS may facilitate recovery through regional synchronization, inter‐regional connectivity, and global network optimization.

Our results suggest that active dual‐tDCS produces significant improvements across multiple cognitive domains compared to sham stimulation, supporting the therapeutic potential of neuromodulation in PSCI recovery [9, 10, 33, 34]. While the DLPFC is a well‐established target, the present study focuses on the implementation of a simultaneous bilateral configuration [35]. Specifically, this framework is designed to modulate the interhemispheric functional imbalance frequently observed secondary to stroke [36], providing a standardized and clinically viable strategy for cognitive rehabilitation. These observed behavioral improvements parallel the functional network modulations captured across distinct imaging metrics.

At the regional level, increased ReHo in the right PHG and left IFG correlated with global cognitive gains. These regions are integral to the neural architecture of episodic memory and attentional control [37, 38]. The significance of ReHo as a reliable indicator of cognitive status is further underscored by our previous cross‐sectional investigation [15], which identified divergent spontaneous activity within the gyrus rectus between PSCI patients and stroke survivors with spared global cognition. This earlier evidence of localized pathological alterations in the deep frontal lobe provides a basis for the current intervention. Furthermore, the importance of these intrinsic regional signatures is reinforced by recent findings identifying regional functional instability as a hallmark of PSCI [39].

While modalities such as repetitive transcranial magnetic stimulation (rTMS) have demonstrated efficacy in modulating cerebral function in PSCI [16], our dual‐tDCS protocol offers greater practical advantages in terms of clinical accessibility. Unlike stationary and resource‐intensive rTMS equipment, the portable and cost‐effective profile of bilateral tDCS facilitates broader integration into community‐based rehabilitation. By identifying the modulation of ReHo within the specific regions highlighted in our previous and current work, the present study links regional functional changes to clinical cognitive improvements. This provides a potential mechanistic insight into how this dual‐tDCS protocol supports cognitive functional recovery in PSCI.

Building upon these regional functional changes, our analysis of distant connectivity explores how localized alterations in brain activity are reflected in broader network‐level interactions. The selection of the DLPFC as the stimulation target is based on its established role as a hub within the executive control network (ECN) [40, 41]. While the observed FC changes did not directly overlap with the stimulation site, such discrepancies between the stimulation site and areas of altered activation are not uncommon in neuromodulation literature [30, 42], as the therapeutic impact of tDCS may propagate through interconnected circuits [43].

On the inter‐regional network level, the reduction in FC between the right MTG and left SFG [key nodes of the default mode network (DMN)] following intervention significantly correlated with improved attentional performance. Such DMN attenuation aligns with evidence that mitigating pathological DMN hyper‐connectivity is a key feature of functional recovery in PSCI [11, 44, 45]. These findings support the competitive interaction between the ECN and DMN, which typically characterizes cognitive processing [46, 47]. Specifically, bilateral DLPFC stimulation may enhance ECN‐mediated activity, which indirectly suppresses task‐irrelevant DMN synchrony through inherent competitive dynamics. This shift in the balance between task‐positive and task‐negative systems likely optimizes neural resource allocation by reducing internal interference during cognitive tasks [24, 48]. Furthermore, the modulation of IFG‐cerebellar connectivity highlights the contribution of the cerebro‐cerebellar axis [49, 50] within a functional modulation of circuits relevant to cognitive control [51].

Beyond regional and inter‐regional modulations, we employed graph theory to evaluate the holistic impact of dual‐tDCS on the topological organization of brain. Stroke typically disrupts the small‐world architecture that balances localized specialization with global integration, leading to increased path lengths and decreased global efficiency [12, 26, 52].

Our findings demonstrate that dual‐tDCS facilitates a transition toward a more efficient small‐world topology, characterized by increased small‐worldness (*σ*), normalized clustering (*γ*), and global efficiency (*E*_glob_), alongside reduced characteristic path length (*L*_p_), which was strongly correlated with improvements in global cognition and attention. These topological shifts are consistent with recent clinical evidence in PSCI populations, where tDCS has been shown to enhance small‐world architecture and network efficiency [13]. This alignment suggests that the restoration of the global network economy is a robust feature of tDCS‐induced cognitive recovery.

These large‐scale changes are mechanistically supported by hybrid brain modeling, which demonstrates that focal modulation of the DLPFC can effectively reset global network efficiency [53]. Findings from this model suggest that modulating the DLPFC, a central hub of the executive control network, promotes a cascade of activity across structural connections, thereby refining the global efficiency and dynamic flexibility of brain. Our results provide empirical support for these predictions, suggesting that the clinical benefits of our approach are associated with a large‐scale optimization of functional network topology.

In summary, our findings across regional (ReHo), circuit (FC), and global (graph theory) levels provide an integrated characterization of the functional network alterations induced by dual‐tDCS. The simultaneous modulation of localized synchronization, inter‐regional connectivity, and large‐scale network organization suggests that tDCS influences the functional architecture across hierarchical levels. These combined findings indicate that the cognitive gains observed in PSCI are associated with functional network efficiency and topological stability, offering empirical neuroimaging evidence for evaluating the clinical efficacy of this intervention.

Beyond these fMRI shifts, the clinical impact of this protocol is further evidenced by the improvements in Barthel Index scores. These observations align with previous findings that tDCS‐induced cognitive gains are often accompanied by enhanced functional independence in post‐stroke populations [9, 10, 33]. Regarding safety, the low incidence of mild, transient adverse effects (tingling erythema) confirms the tolerability of the 2.0 mA dual‐tDCS protocol [34, 54]. The absence of serious adverse events reinforces the feasibility of this intervention, supporting its potential for broader implementation in clinical rehabilitation settings.

Given this favorable safety profile and positive impact on daily functioning, transitioning these empirical findings into routine clinical practice becomes highly feasible. This trial bridges the gap between advanced imaging and clinical neurorehabilitation. Bilateral DLPFC dual‐tDCS offers a safe, low‐cost, and non‐invasive adjunct easily integrated into clinical bedside workflows, community rehabilitation, or supervised home‐based settings for patients with persistent cognitive impairment who are at least 3 months after stroke onset. Crucially, resting‐state fMRI functions to monitor the clinical effects of neuromodulation by capturing alterations spanning specific regions, inter‐regional networks, and global efficiency. These functional network changes offer a promising theoretical foundation for future precision interventions, establishing these imaging metrics as potential biological markers to guide rehabilitation tailored to individual network profiles.

## 5 Limitations

Several limitations warrant consideration. First, while significant effects were observed, the relatively modest sample size and the lack of stratification by stroke subtype or baseline severity may constrain the generalizability of these findings. Future large‐scale, multicenter trials are necessary to validate these results across a more diverse range of clinical phenotypes. Furthermore, the lack of long‐term follow‐up precludes an assessment of the durability of the observed functional network alterations; therefore, longitudinal studies with extended follow‐up periods are required to determine the persistence of these therapeutic effects.

Second, our imaging analysis was restricted to resting‐state functional networks. Future research should adopt a multimodal imaging approach, integrating diffusion tensor imaging (DTI) to investigate the coupling between structural white matter integrity and functional network organization. Additionally, task‐based fMRI could provide insights into how these resting‐state alterations translate into dynamic neural recruitment during specific cognitive challenges.

Finally, despite rigorous clinical screening, the absence of AD‐related biomarkers represents a limitation in fully excluding mixed neurodegenerative pathologies. Future investigations utilizing biomarker‐confirmed cohorts will be instrumental in refining the specificity of tDCS effects in post‐stroke populations.

## 6 Conclusions

Bilateral DLPFC dual‐tDCS is a safe and effective intervention for improving global cognition, attention, memory, and functional independence in PSCI patients. These clinical benefits are associated with distinct rs‐fMRI alterations, including regional synchronization in the IFG, inter‐regional connectivity between the ECN and DMN (including cerebellar networks), and enhanced global network efficiency. By these hierarchical modifications, our findings suggest a potential neuroimaging biomarker for monitoring tDCS efficacy, offering a solid rationale for precision neuromodulation in stroke rehabilitation across clinical, community, and supervised home‐based settings.

## Funding

This study was funded by the Nanjing Municipal Special Fund Key Project for Health Science and Technology Development (ZKX22042), and the Nanjing Municipal Special Fund General Project for Health Science and Technology Development (YKK22137).

## Ethics Statement

The Nanjing Medical University Affiliated Brain Hospital ethics committee approved this study (No. 2022‐KY086‐01, Approval Date: March 29th, 2022). All participants gave written informed consent.

## Conflicts of Interest

The authors declare no conflicts of interest.

## Supporting information


**Data S1:** CONSORT 2010 checklist of information to include when reporting a randomized trial. Medication clarification, Routine Cognitive Function Training, and Brain MRI Data Acquisition.
**Figure S1:** The 116AAL atlas.
**Table S1:** Characteristics at baseline for the tDCS and the sham groups fulfilled fMRI.
**Figure S2:** Lesion distribution maps of the participants.

## Data Availability

The data that support the findings of this study are available from the corresponding author upon reasonable request.
