# Supplementary material for: Dual Transcranial Direct Current Stimulation Modulates Hierarchical Functional Network Organization in Post‐Stroke Cognitive Impairment: A Randomized Controlled Trial
Source: CNS Neurosci Ther. 2026 Sep 2;32(9):e71111. doi: 10.1002/cns.71111 (PMC13538461; doi:10.1002/cns.71111)
Supplement: Supplementary file 1 [file CNS-32-e71111-s001.docx]

**Supplementary Material File**

CONSORT 2010 checklist of information to include when reporting a randomised trial*

| Section/Topic | Item No | Checklist item | Reported on page No |
| --- | --- | --- | --- |
| Title and abstract | | | |
|  | 1a | Identification as a randomised trial in the title | 1 |
|  | 1b | Structured summary of trial design, methods, results, and conclusions (for specific guidance see CONSORT for abstracts) | 2-3 |
| Introduction | | | |
| Background and objectives | 2a | Scientific background and explanation of rationale | 3-4 |
|  | 2b | Specific objectives or hypotheses | 4 |
| Methods | | | |
| Trial design | 3a | Description of trial design (such as parallel, factorial) including allocation ratio | 4-5 |
|  | 3b | Important changes to methods after trial commencement (such as eligibility criteria), with reasons | 4-5 |
| Participants | 4a | Eligibility criteria for participants | 5 |
|  | 4b | Settings and locations where the data were collected | 5 |
| Interventions | 5 | The interventions for each group with sufficient details to allow replication, including how and when they were actually administered | 5-6 |
| Outcomes | 6a | Completely defined pre-specified primary and secondary outcome measures, including how and when they were assessed | 6-8 |
|  | 6b | Any changes to trial outcomes after the trial commenced, with reasons | 6-8 |
| Sample size | 7a | How sample size was determined | 5 |
|  | 7b | When applicable, explanation of any interim analyses and stopping guidelines | 5-8 |
| Randomisation: |  |  |  |
| Sequence generation | 8a | Method used to generate the random allocation sequence | 4-5 |
|  | 8b | Type of randomisation; details of any restriction (such as blocking and block size) | 4-5 |
| Allocation concealment mechanism | 9 | Mechanism used to implement the random allocation sequence (such as sequentially numbered containers), describing any steps taken to conceal the sequence until interventions were assigned | 4-5 |
| Implementation | 10 | Who generated the random allocation sequence, who enrolled participants, and who assigned participants to interventions | 4-5 |
| Blinding | 11a | If done, who was blinded after assignment to interventions (for example, participants, care providers, those assessing outcomes) and how | 4-5 |
|  | 11b | If relevant, description of the similarity of interventions |  |
| Statistical methods | 12a | Statistical methods used to compare groups for primary and secondary outcomes | 8 |
|  | 12b | Methods for additional analyses, such as subgroup analyses and adjusted analyses | 8 |
| Results | | | |
| Participant flow (a diagram is strongly recommended) | 13a | For each group, the numbers of participants who were randomly assigned, received intended treatment, and were analysed for the primary outcome | 8-9, Figure 1 |
|  | 13b | For each group, losses and exclusions after randomisation, together with reasons | 8-10, Figure 1 |
| Recruitment | 14a | Dates defining the periods of recruitment and follow-up |  |
|  | 14b | Why the trial ended or was stopped |  |
| Baseline data | 15 | A table showing baseline demographic and clinical characteristics for each group | 21, Table 1 |
| Numbers analysed | 16 | For each group, number of participants (denominator) included in each analysis and whether the analysis was by original assigned groups | 8-10, Figure1 |
| Outcomes and estimation | 17a | For each primary and secondary outcome, results for each group, and the estimated effect size and its precision (such as 95% confidence interval) | 9-11, Figure 2-6, Table 2-3 |
|  | 17b | For binary outcomes, presentation of both absolute and relative effect sizes is recommended | - |
| Ancillary analyses | 18 | Results of any other analyses performed, including subgroup analyses and adjusted analyses, distinguishing pre-specified from exploratory | - |
| Harms | 19 | All important harms or unintended effects in each group (for specific guidance see CONSORT for harms) | 11 |
| Discussion | | | |
| Limitations | 20 | Trial limitations, addressing sources of potential bias, imprecision, and, if relevant, multiplicity of analyses | 14-15 |
| Generalisability | 21 | Generalisability (external validity, applicability) of the trial findings | 11-14 |
| Interpretation | 22 | Interpretation consistent with results, balancing benefits and harms, and considering other relevant evidence | 11-14 |
| Other information | | |  |
| Registration | 23 | Registration number and name of trial registry | 5 |
| Protocol | 24 | Where the full trial protocol can be accessed, if available | - |
| Funding | 25 | Sources of funding and other support (such as supply of drugs), role of funders | 16 |

*We strongly recommend reading this statement in conjunction with the CONSORT 2010 Explanation and Elaboration for important clarifications on all the items. If relevant, we also recommend reading CONSORT extensions for cluster randomised trials, non-inferiority and equivalence trials, non-pharmacological treatments, herbal interventions, and pragmatic trials. Additional extensions are forthcoming: for those and for up to date references relevant to this checklist, see [www.consort-statement.org](http://www.consort-statement.org).

**Medication clarification**

All participants received standardized medical management for stroke secondary prevention and comorbidity control in accordance with current clinical guidelines. This included antiplatelet or anticoagulant therapy (e.g., aspirin, clopidogrel, or warfarin), lipid-lowering agents (primarily statins such as atorvastatin and rosuvastatin), and antihypertensive or hypoglycemic medications (e.g., ACEI/ARB, CCB, metformin, and insulin) according to current clinical guidelines^[1]^. To minimize confounding effects on cognitive outcomes, medications aimed at improving cerebral blood flow or neuroprotection (e.g., butylphthalide and neurotrophic agents) were monitored. Specifically, for cognition-related drugs (e.g., oxiracetam, donepezil)^[2]^, inclusion required patients to be on a stable dosage for at least four weeks prior to enrollment. All participants-maintained baseline medication regimens without dosage titration or pharmacological changes throughout the 4-week intervention period.

**Routine Cognitive Function Training**

Despite the lack of high-quality evidence to support it^[2]^, we still incorporated the cognitive function training, or cognitive skills training into our routine management for all the PSCI participants. The detailed cognitive training elements comprise: 1) Orientation training: Employing personal artifacts to elicit significant times, locations, and individuals; 2) Attention training: Improving concentration with deletion puzzles, connect-the-dots activities, and mini-games; 3) Memory training: Numerical and visual memory capacity, working memory (delayed retrieval of images, faces, etc.); 4) Calculation Training: Improving numerical proficiency using mathematical games and virtual retail experiences; 5) Visuospatial Perception Training: Engaging in puzzle and tangram activities; 6) Logical Reasoning Training: Enhancing inferential skills through visual reasoning and comic strip sequencing.

**Brain MRI Data Acquisition**

MRI scanning was acquired with a Siemens 3.0-T signal scanner (Siemens, Verio, Germany) in Affiliated Brain Hospital of Nanjing Medical University. All subjects lay supine with their head fixed by foam pads with a standard birdcage head coil to minimize head movement. Participants were instructed to remain as still as possible, open their eyes, remain awake, and not think of anything.

For functional analysis and localization, we acquired:

1. High-resolution T1-weighted images were acquired by 3D magnetization-prepared rapid gradient-echo (MPRAGE) sequence (repetition time [TR] =1900ms; echo time [TE] =2.48ms; flip angle [FA] =9 degrees; matrix =256×256; field of view [FOV] =256×256 mm^2^ ; slice thickness = 1.0 mm; no gap; 176 slices covered the whole brain) for image registration and functional localization. The imaging took approximately 266 seconds.

2. Functional images were subsequently collected in the same slice orientation with a gradient-recalled echo-planar imaging pulse sequence (TR= 2,000ms; TE =30ms; FA =90degrees; matrix= 64×64, FOV= 220×220mm^2^; thickness/gap =4.0/0mm; voxel size = 3.4×3.4×4 mm^3^; slice numbers =36). A total of 240 volumes were obtained in this acquisition sequence and each functional resting-state session lasted 480 seconds.

For routine structural assessment and lesion characterization, the following sequences were performed:

1. Axial T2-weighted images were acquired by a 2D turbo spin-echo sequence with BLADE motion correction (repetition time [TR] = 3330 ms; echo time [TE] = 106 ms; flip angle [FA] = 150 degrees; matrix = 384 × 384; field of view [FOV] = 220 × 220 mm²; slice thickness/gap = 6.0/1.8 mm; 16 slices covered the target brain regions) for structural evaluation and lesion identification. The imaging took approximately 77 seconds.

2. Axial T2-weighted fluid-attenuated inversion recovery (FLAIR) images were acquired by a 2D turbo inversion recovery magnitude (TIRM) sequence (repetition time [TR] = 8000 ms; echo time [TE] = 81 ms; inversion time [TI] = 2373.3 ms; flip angle [FA] = 150 degrees; matrix = 230 × 208; field of view [FOV] = 230 × 208 mm²; slice thickness/gap = 6.0/1.8 mm; 16 slices covered the target brain regions) for the assessment of white matter hyperintensities and lesions. The imaging took 96 seconds.

3. Axial diffusion-weighted images (DWI) were acquired by a readout-segmented echo-planar imaging (RESOLVE) sequence (repetition time [TR] = 2880 ms; echo time [TE] = 64 ms; flip angle [FA] = 180 degrees; b-values = 0 and 1000 $s/mm^2$; 3 diffusion directions; matrix = 160 × 160; field of view [FOV] = 220 × 220 mm²; slice thickness/gap = 6.0/1.8 mm; 16 slices covered the target brain regions) for the assessment of water diffusion and lesion detection. The imaging took 63 seconds.

**Supplementary Figure 1. The 116AAL atlas**

**Supplementary Table 1. Characteristics at baseline for the tDCS and the sham groups fulfilled fMRI**

| **Characteristic** | **tDCS group** | **Sham group** | ***P* value** |
| --- | --- | --- | --- |
|  | **(n=18)** | **(n=18)** |  |
| Gender (male/female), n | 12/6 | 15/3 | 0.248 |
| Age (years), Mean ± SD | 57.11±13.84 | 57.83±12.16 | 0.869 |
| Education, n |  |  | 0.118 |
| ≤ 9 years | 6 | 2 |  |
| > 9 years | 12 | 16 |  |
| Duration (days), Mean ± SD | 101.56±11.24 | 115.78±29.38 | 0.063 |
| Stroke type (ischemia/hemorrhage), n | 11/7 | 11/7 | 1.000 |
| Side of lesion (left/right), n | 11/7 | 10/8 | 0.735 |
| Lesion volume (cc) | 8.08±8.46 | 9.58±13.20 | 0.687 |
| Site of lesion, n |  |  | 0.711 |
| Cortico-Subcortical Strokes | 12 | 10 |  |
| Deep Hemispheric WM and GM Strokes | 3 | 3 |  |
| Brainstem/Cerebellar Strokes | 3 | 5 |  |
| NIHSS level, Median (IQR) ^a^ | 3.00 (3.00-3.25) | 3.00 (3.00-3.00) | 0.609 |
| Fazekas score (0/1)^[3]^, n | 3/15 | 4/14 | 0.674 |
| MoCA Mean ± SD | 15.61±3.29 | 16.78±3.89 | 0.338 |
| BI, Mean ± SD | 50.83±22.11 | 57.50±22.11 | 0.372 |

BI：Barthel Index; GM: Gray Matter; IQR: Interquartile Range; MoCA: Montreal Cognitive Assessment; NIHSS: National Institute of Health stroke scale; SD: Standard Deviation; tDCS: Transcranial Direct Current Stimulation; WM: White Matter.

^a^ NIHSS scores were categorized into 5 levels: Level 1 (0-1), Level 2 (2-4), Level 3 (5-15), Level 4 (16-20), and Level 5 (≥21).

**Supplementary Figure 2. Lesion distribution maps of the participants**. The figure illustrates the lesion distribution for patients with left-sided lesions (top row) and right-sided lesions (bottom row). The color scale represents the frequency of lesion overlapping at each voxel across subjects. The colors range from purple (minimum overlap, n = 1) to red/yellow (maximum overlap), indicating the number of patients with a lesion at a specific anatomical location. Lesions were manually delineated on individual T1-weighted images and normalized to the Montreal Neurological Institute (MNI) space ^[4]^. The sagittal views on the right indicate the levels of the axial slices shown.

References:

[1] Kleindorfer D O, Towfighi A, Chaturvedi S, et al. 2021 Guideline for the Prevention of Stroke in Patients With Stroke and Transient Ischemic Attack: A Guideline From the American Heart Association/American Stroke Association[J]. Stroke, 2021, 52(7): e364-e467.

[2] Quinn T J, Richard E, Teuschl Y, et al. European Stroke Organisation and European Academy of Neurology joint guidelines on post-stroke cognitive impairment[J]. European stroke journal, 2021, 6(3): I-XXXVIII.

[3] Fazekas F, Chawluk J B, Alavi A, et al. MR signal abnormalities at 1.5 T in Alzheimer's dementia and normal aging[J]. AJR. American journal of roentgenology, 1987, 149(2): 351-356.

[4] A. C. Evans, D. L. Collins, S. R. Mills, et al. 3D statistical neuroanatomical models from 305 MRI volumes.3D statistical neuroanatomical models from 305 MRI volumes. In: 1993 IEEE Nuclear Science Symposium and Medical Imaging Conference. IEEE; 1993:1813-1817.
